# The Homeobox Gene Gsx2 Regulates the Self-Renewal and Differentiation of Neural Stem Cells and the Cell Fate of Postnatal Progenitors

**DOI:** 10.1371/journal.pone.0029799

**Published:** 2012-01-05

**Authors:** Héctor R. Méndez-Gómez, Carlos Vicario-Abejón

**Affiliations:** 1 Instituto Cajal, Consejo Superior de Investigaciones Científicas (CSIC), Madrid, Spain; 2 Centro de Investigación Biomédica en Red sobre Enfermedades Neurodegenerativas (CIBERNED), Madrid, Spain; VIB & Katholieke Universiteit Leuven, Belgium

## Abstract

The Genetic screened homeobox 2 (Gsx2) transcription factor is required for the development of olfactory bulb (OB) and striatal neurons, and for the regional specification of the embryonic telencephalon. Although Gsx2 is expressed abundantly by progenitor cells in the ventral telencephalon, its precise function in the generation of neurons from neural stem cells (NSCs) is not clear. Similarly, the role of Gsx2 in regulating the self-renewal and multipotentiality of NSCs has been little explored. Using retroviral vectors to express Gsx2, we have studied the effect of Gsx2 on the growth of NSCs isolated from the OB and ganglionic eminences (GE), as well as its influence on the proliferation and cell fate of progenitors in the postnatal mouse OB. Expression of Gsx2 reduces proliferation and the self-renewal capacity of NSCs, without significantly affecting cell death. Furthermore, Gsx2 overexpression decreases the differentiation of NSCs into neurons and glia, and it maintains the cells that do not differentiate as cycling progenitors. These effects were stronger in GESCs than in OBSCs, indicating that the actions of Gsx2 are cell-dependent. *In vivo*, Gsx2 produces a decrease in the number of Pax6^+^ cells and doublecortin^+^ neuroblasts, and an increase in Olig2^+^ cells. In summary, our findings show that Gsx2 inhibits the ability of NSCs to proliferate and self-renew, as well as the capacity of NSC-derived progenitors to differentiate, suggesting that this transcription factor regulates the quiescent and undifferentiated state of NSCs and progenitors. Furthermore, our data indicate that Gsx2 negatively regulates neurogenesis from postnatal progenitor cells.

## Introduction

Transcription factors are crucial to generate all classes of neurons and glial cells, both during development and adulthood. The spatial and temporal regulation of transcription factors controls regional identity in the central nervous system (CNS), as well as the self-renewal, proliferation and fate of neural stem and progenitor cells [1], [2], [3]. Olfactory bulb (OB) neurons originate from molecularly defined, spatially distinct proliferative regions. Glutamatergic projection neurons are generated during the embryonic period in the local OB ventricular zone (VZ), a territory in the dorsal/pallial telencephalon in which Pax6 is expressed [4], [5], [6], [7]. Embryonic OB interneurons are derived from the distal Gsx2 and Er81 expressing cells in the lateral ganglionic eminence (LGE) of the ventral/subpallial telencephalon [8], [9], and from progenitors outside the LGE, including the OB itself [7], [10], [11], [12]. Postnatally, interneurons arise from the subventricular zone (SVZ) of the lateral ventricle [13], [14], [15], although the rostral migratory stream (RMS) and the OB are also thought to serve as a source of neurons [16], [17], [18].

From the early stages of telencephalic development, the pallium and subpallium can be identified by the expression of the Paired Type Homeobox 6 (Pax6) and the Genomic Screened Homeobox 2 (Gsx2; formerly Gsh2) transcription factors, respectively. Gsx2 expression in the LGE [19], [20] has been implicated in the control of the pallium-subpallium boundary by repressing the expression of dorsal genes, such as *Pax6*. Indeed, deletion of the *Gsx2* gene causes an expansion of Pax6 expression into the LGE [9], [21], [22], [23] whereas Gsx2 overexpression in transgenic mice reduces the expression of pallial markers in the embryonic telencephalon [9].

In addition to its role in dorso-ventral patterning, Gsx2 is thought to be necessary for the formation and/or maintenance of striatal projection neurons and OB interneurons [9], [22], [24], [25]. However, a direct positive effect of Gsx2 on interneuron generation was only described for a subpopulation of cortical calretinin neurons [26] and not for OB interneurons. Although defects in progenitor cell proliferation and expansion have been described in Gsx2 knockout mice [21], [25], [27], the specific role of this transcription factor in these processes has not been investigated in depth. Gsx2 is thought to affect Notch signaling in the LGE [28], which influences self-renewal and gliogenesis [3]. Moreover, recent data indicates that high levels of Gsx2 may maintain LGE progenitors in a dividing and undifferentiated state [29]. By contrast, enhanced proliferation has been described in the cerebral cortex of Gsx2 mutants [30].

In the present study we sought to investigate the role of Gsx2 during the development of the OB and GE through gain-of-function experiments using retroviral vectors to achieve sustained Gsx2 expression. This system allows us to analyze the effects of Gsx2 at the single-cell level. Accordingly, we analyzed the effects of Gsx2 in the self-renewal, proliferation and differentiation of NSCs isolated from the OB and GE, and in the proliferation and cell fate of postnatal OB progenitors *in vivo*. We found that sustained Gsx2 expression diminished the potential of cultured NSCs to self-renew and differentiate, and it favored the maintenance of NSC-derived progenitors in the cell cycle. Moreover, Gsx2 overexpression reduced the capacity of postnatal progenitors to differentiate into neurons in vivo. Our findings suggest a new role for Gsx2 in regulating the self-renewal and differentiation of NSCs, and in determining the cell fate of postnatal progenitors.

## Methods

### Ethical statement

All animal care and handling was carried out in accordance with European Union guidelines (directive 86/609/EEC) and was approved by the Comisión de Bioética (Ethical Committee) of the Consejo Superior de Investigaciones Científicas (CSIC: certificates SAF2004-05798 issued on January 23, 2004; GR/SAL/0835/2004 issued on June 10, 2004; and BFU2007-61230 issued on June 7, 2007). All efforts were made to ameliorate the suffering of the animals.

### Neural stem cell (NSC) cultures

NSCs were prepared from the OB and GE of embryonic day 13.5 (E13.5) CD1 mice [2], [7], and from the OB of postnatal day 4 (P4) mice. To obtain brain tissue, pregnant mice were killed by cervical dislocation and the embryos were then decapitated, while P4 mice were placed on ice and decapitated. After dissociating the tissue obtained, the cells were plated and expanded as neurospheres by daily addition of 20 ng/ml fibroblast growth factor-2 (FGF-2; PeproTech) and 20 ng/ml epidermal growth factor (EGF; PeproTech) in Dulbecco's modified Eagle medium (DMEM)/nutrient mixture F12 (F12), supplemented with insulin, apotransferrin, putrescine, progesterone and sodium selenite (N2: DMEM/F12/N2). Most experiments were performed with cells between passages 3–10, when the majority of cells exhibit a stable karyotype [18].

### Cloning, subcloning, virus production and infection of NSCs with retroviral vectors

Infection was achieved using a Moloney murine leukaemia virus-based retroviral vector (Retrofect®: Cellerix S.L, Madrid [2]). The retroviral construct pRV-IRES-EGFP (referred to simply as the EGFP vector) was used as a control in infection experiments. The human *Gsx2 ORF* (1 Kb) was amplified from a pcDNA-Gsh2 plasmid [31] by PCR using specific 5′ and 3′ primers containing BamHI and XhoI restriction sites (sense primer: 5′-TTG GGA TCC ACC ATG TCG CGC TCC TTC TAT G-3′; antisense primer: 5′-TTC CTC GAG TCA TAA GGG GGA AAT CTC CTT-3′). The PCR fragment amplified was cloned into the pRV-IRES-EGFP vector and then sequenced. A clone was selected in which there were no mutations in the coding region and this was then used to generate viral particles by transfecting it into 293T HEK cells. The titers obtained were in the range of 10^5^–10^6^ infection units (IU)/ml, as measured by infecting NIH 3T3 cells with increasing volumes of supernatant and then performing flow cytometry analysis. When the supernatant containing the viral particles was concentrated by ultracentrifugation, the resulting titers were in the range of 10^7^ IU/ml. Concentrated viral particles were used in all the *in vivo* experiments whereas in experiments performed on NSC cultures, non-concentrated and concentrated viral particles were used. To calculate the efficiency of infection in neural cells, proliferating NSCs were harvested and analyzed by flow cytometry to determine the proportion of cells expressing EGFP.

To infect proliferating NSCs, 3.5×10^6^ cells were dissociated and resuspended in culture medium, and then incubated in the presence of the viral supernatant and 6 µg/ml of polybrene. Subsequently, the cells were collected, seeded at a density of 15,000 cells/cm^2^, and then incubated in the presence of FGF-2 and EGF. OBSC cultures contained up to 55.2% of GFP^+^ cells when infected with the Gsx2-EGFP vector (not shown), whereas the efficiency of infection with the EGFP vector was up to 96.6%, as described previously [2]. To obtain cultures that contained the same ratio of infected and uninfected cells, fewer EGFP particles than Gsx2-EGFP particles were added to the cultures. Moreover, since the results obtained with cultures that had different infection efficiencies were indistinguishable, they were combined in the analysis. At maximal EGFP expression (3–4 days after infection), NSCs were passaged and plated for an additional 3–4 days on polyornithine-coated coverslips under conditions that promote proliferation (5,000–6,000 cells/cm^2^ with FGF-2 and EGF). Proliferating cells were incubated for 20 hours with 5 µM 5′-bromo-2-deoxyuridine (BrdU: Roche Diagnostics), a dose previously shown to be non-toxic for NSC proliferation [2], and they were then fixed with 4% paraformaldehyde (PFA).

For cell differentiation studies, neurospheres were disaggregated and plated at a density of 100,000 cells/cm^2^ in DMEM/F12/N2 plus 5% FBS for 6–20 days. A single dose of FGF-2 (20 ng/ml) was added on the first day of plating, which enhanced neuronal differentiation of the NSCs [18], although it did not affect Gsx2 overexpression (data not shown). Cells were finally fixed with 4% PFA and immunostained.

For the clonal analysis, E13.5 OBSC primary cell neurospheres were mechanically dissociated and resuspended at a concentration of 1 cell/100 µl of DMEM/F12/N2∶OBSC-conditioned medium (1∶1), of which 200 µl was placed in each well of 96-well plates. The following day, wells containing a single EGFP^+^ cell were marked and proliferation was induced for 9 days. Clonally-derived neurospheres were fixed and photographed under phase contrast and fluorescence optics. The cell diameter of the clonal neurospheres expressing EGFP was measured using ImageJ software (Wayne Rasband, National Institutes of Health, USA). The results are expressed as the mean ± s.e.m and statistical analyses were performed using a two-tailed Student's *t* test. In all the experiments, EGFP and Gsx2-EGFP infection was carried out and analyzed in parallel.

### Immunostaining of cultured cells

Cells were incubated overnight at 4°C or Room Temperature with primary antibodies raised against: GFP (mouse, rabbit and rat antibodies, 1∶1,000–1∶2,000: Molecular Probes/Invitrogen, Eugene, OR, Cat. n. A-11120 and A-6455, and Nacalai Tesque, Kyoto, Japan, 04404-84), Gsx2 (rabbit, 1∶2,000–1∶4,000: kindly provided by K. Campbell, University of Cincinnati, OH), β-III-tubulin (TuJ1, rabbit, and mouse antibodies, 1∶1,000–1∶2,000: Covance, Berkeley, CA, Cat. n. PRB-435P and MMS-435P); GFAP (rabbit polyclonal, 1∶1,000: Dako, Glostrup, Denmark, Cat. n. Z0334); O4 (mouse monoclonal IgM, 1∶8 obtained from the culture media of O4-producing hybridoma cells: kindly provided by A. Rodríguez-Peña, CSIC, Madrid, Spain); Nestin (rabbit polyclonal, 1∶3,000: kindly provided by R. McKay, National Institutes of Health, Bethesda, MD); BrdU (mouse antibodies, 1∶1,000 and 1∶100: clone G3G4, the Developmental Studies Hybridoma Bank, University of Iowa Dept. Biological Sciences, Iowa City, IA, and Becton Dickinson, San Jose, CA, 7580); Ki67 (mouse monoclonal, 1.500: Dako, Cat. n. M7248). The cells were then incubated with Alexa fluor 488 or 594 conjugated secondary antibodies (1∶400–500; Invitrogen) and finally with 4′,6-diamidino-2-phenylindole (DAPI, Vector Laboratories, Burlingame, CA), before mounting in Mowiol solution (Calbiochem, San Diego, CA). Controls were performed to confirm the specificity of the primary and secondary antibodies.

### Cell death assays

Three different procedures were used to assay the cell death in the cultures.

1. Caspase 3 immunostaining. For this purpose, proliferating cells growing on polyornithine-coated coverslips (in adherent conditions) were fixed directly, whereas neurospheres were first collected on matrigel and then fixed with PFA. Samples were incubated overnight at 4°C with primary antibodies raised against GFP and cleaved caspase 3 (Asp175; rabbit, 1∶400: Cell Signaling, Danvers, MA, USA, Cat. n. 9661).
2. The In Situ Cell Death Detection Kit (TMR red, Roche, Mannheim, Germany) was used to detect apoptosis in cells immunostained with anti-GFP antibodies. Cells were incubated with BGT (3 mg/ml BSA, 100 mM glycine, 0.25% Triton X-100) at 37°C for 30 minutes, and then for 1 hour with a solution containing terminal deoxynucleotidyl transferase and fluorescent labeled dNTP at a ratio of 1∶10.
3. The APC Annexin V kit (BD Pharmingen, Cat. n. 561012) was used in conjunction with propidium iodide to detect cells undergoing early and late apoptosis following the manufacturer's instructions with some modifications. Briefly, cells were washed with cold PBS, incubated with APC Annexin V in binding buffer and then, propidium iodide was added to the cells that were finally analyzed by flow cytometry.

### Immunoblotting

Cell extracts from proliferating EGFP and Gsx2-EGFP E13.5 OBSC cultures were analyzed in immunoblots probed with primary antibodies against Gsx2 and β-tubulin (1∶1,000: Sigma, Cat. n. T4026). The optical density of the specific protein bands was measured by densitometry using Quantity One software (Bio-Rad, Madrid, Spain) to estimate the relative protein levels. The majority of densitometry signals were not saturated and when saturation did occur, those pixels were highlighted by the detector and eliminated from the analysis. Gsx2 levels were normalized to those of β-tubulin.

### Reverse transcriptase–polymerase chain reaction (RT-PCR)

Total RNA was extracted from E13.5 OBSCs using the TRIZOL Reagent (Invitrogen) according to the manufacturer's instructions. Contaminating DNA was removed from 2 µg of RNA by treatment with deoxyribonuclease I (DNaseI) and this enzyme was then inactivated with EDTA. Reverse transcription (RT) was carried out using SuperScript III (Invitrogen) and to detect specific complementary DNAs (cDNAs), PCR reactions were performed using specific primers described elsewhere [18].

### *In vivo* injection of retroviral vectors expressing EGFP and Gsx2-EGFP

Concentrated viral particles (1 µl) were injected into the subependymal zone (SEZ) of the OB of postnatal day 4 (P4) mice using a glass pipette connected to an injection kit (World Precision Instruments), which was in turn connected to a Hamilton pipette. Animals were anesthetized by placing them in ice for 2 minutes and they were then positioned in a mouse neonatal adaptor (Stoelting) fixed to a standard stereotaxic apparatus (Kopf). The stereotaxic coordinates for the SEZ were: anteroposterior to bregma +1.3 mm, lateral to midline 0.6, ventral to dura 0.5 mm [2]. The mice were then allowed to recover on a heated pad and returned to their mother. The mice were anesthetized 3 or 5 days post-injection, and then perfused transcardially with 0.9% NaCl and 4% PFA. Their brains were post-fixed, embedded in 3% agarose and serial coronal 50 µm vibratome sections were obtained. Sections containing the OB were collected and immunostained with antibodies against GFP, Gsx2, Phospho-Histone H3 (PH-H3, rabbit polyclonal 1∶1,000: Upstate, Cat. n. 06-570), Ki67 (rabbit monoclonal, 1∶1,000: Thermo Sci., Fremont, CA, Cat. n. RM-9106); Olig2 (rabbit polyclonal 1∶2,000: Millipore, Cat. n. AB9610); doublecortin (Dcx, guinea pig polyclonal 1∶3,000: Millipore, Cat. n. AB2253) and Pax6 (rabbit polyclonal 1∶300: Covance, Cat. n. PRB-278P). In all experiments, EGFP and Gsx2-EGFP injections were performed in parallel, as was the analysis of the injected mice.

### Cell counts and statistical analysis

To determine the number of cultured cells expressing a specific antigen, 10 random fields per coverslip were counted using a 40× objective and fluorescence filters (Leica microscope). The number of TdT-mediated dUTP nick-end labeled (TUNEL)^+^ cells was counted in a similar manner. The proportion of cells positive for specific markers and co-labeled with GFP (as determined by immunostaining) was calculated with respect to the total number of GFP-expressing cells or the number of DAPI labeled cells, and the results were expressed as the mean ± s.e.m. of cells in 10 fields taken from 3 to 8 cultures in 2–4 experiments. Statistical analyses were performed using a two-tailed Student's *t* test with Welch's correction when the F test indicated that the variances of both groups differed significantly. The differences between the mean values were considered significant when *P<0.05 (GraphPad Prism 4 software).

In the *in vivo* experiments, the proportion of Gsx2^+^, PH-H3^+^, Ki67^+^, Pax6^+^, Dcx^+^ or Olig2^+^ cells co-labeled with anti-GFP was calculated by counting the cells in animals injected with the EGFP and Gsx2-EGFP expression vectors (n = 3–5 animals per condition). Confocal images were taken from 1–2 microscopy fields from each of three sections per P7 animal and from 5–6 microscopy fields per P9 animal. The entire z-stack of images were then analyzed and the cells were counted manually using ImageJ software (NIH, Bethesda; MD), as described previously [2]. Statistical analyses were performed using the Student's *t* test.

## Results

### Successful overexpression of Gsx2 in neural stem cells

**Figure 1:**
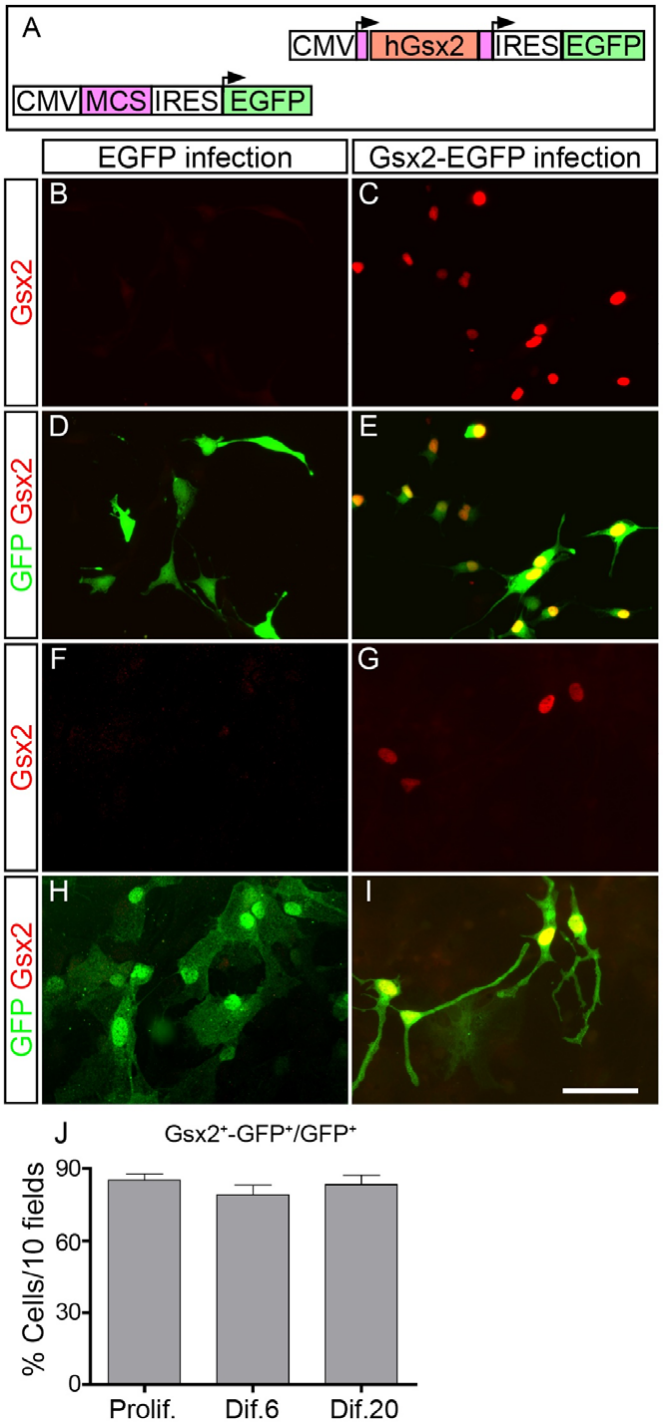
Gsx2 can be efficiently transduced in neural stem cells (NSCs) using retroviral vectors. A) Schematic representation of the constructs used to express Gsx2 and EGFP in NSCs and progenitor cells, both in culture and *in vivo*. B–I) Cells isolated from the OB of E13.5 mice were cultured and passaged as floating neurospheres in the presence of FGF-2 and EGF, and they were then infected with the corresponding viral supernatants. Gsx2^+^ cells were not observed in EGFP-infected OBSC cultures growing under conditions of proliferation (B, D) or differentiation (F, H). By contrast, the majority of GFP^+^ cells (detected by immunostaining) in the Gsx2-EGFP infected cultures expressed Gsx2 while growing under proliferation (85.24%±2.62; Prolif.: C, E, J) or differentiation conditions (for at least 20 days in the presence of 5% FBS, 83.27%±3.90, Dif.: G, I, J). The graph in J represents the mean percentage ± s.e.m. (n = 3 cultures per condition) of cells expressing both Gsx2 and GFP. Scale bar (shown in I) = 60.5 µm.

To determine whether Gsx2 overexpression influences the behavior of neural stem cells (NSCs) during self-renewal, proliferation and differentiation, we performed gain-of-function experiments by introducing retroviral vectors into cultured E13.5 OBSCs, cells that express little Gsx2 mRNA and protein (see below). In addition, we tested the effects of Gsx2 on NSCs isolated from the E13.5 GE, which express high levels of Gsx2 in vivo [7], and those from the P4 OB, mice the same age as those that received the viral injections in vivo (see below). A retroviral vector was prepared by cloning the human *Gsx2* ORF into the pRV-IRES-EGFP plasmid, from which retroviral particles were then produced (Figure 1 A).

The overexpression of Gsx2 protein in E13.5 OBSC cultures grown under proliferation and differentiation conditions was evaluated by dual immunocytochemistry for EGFP and Gsx2. While none of the GFP^+^ cells expressed Gsx2 in EGFP-infected cultures (Figure 1 B, D, F, H), most of the GFP^+^ cells in Gsx2-EGFP cultures were positive for Gsx2 in conditions of proliferation (85.24%±2.62, n = 3: Figure 1 C, E, J). Similar protein co-expression was observed in differentiation conditions (79.12%±4.04, n = 3 at 6 days, short-term differentiation: Figure 1 J) and this was maintained for at least 20 days after the initiation of OBSC differentiation (83.27%±3.90, n = 3: Figure 1 G, I, J). Overexpression of Gsx2 was also confirmed in Western blots and by RT-PCR analysis of proliferating cells (Figure S1).

### Gsx2 overexpression alters the self-renewal capacity of E13.5 OBSCs

**Figure 2:**
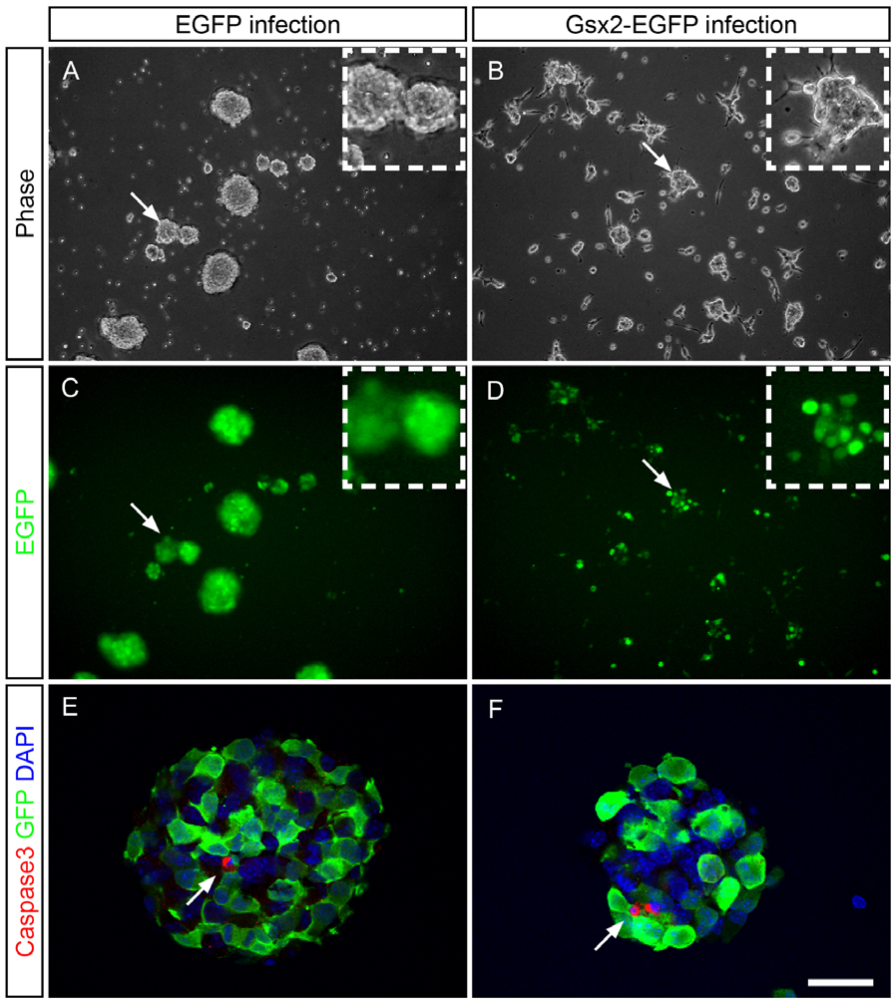
Gsx2 overexpression alters the normal growth of cells as neurospheres. E13.5 OBSCs infected with viral particles expressing EGFP and Gsx2-EGFP were grown under conditions of proliferation with FGF-2 and EGF. Low magnification phase contrast (A, B) and fluorescence microscopy (C, D) images show the cultures 4 days after infection. The images reveal the altered growth of the Gsx2-EGFP-infected neurospheres, which remained relatively small. Some neurospheres attached to the plastic dish and individual cells appeared to leave the neurospheres (B, D). This phenotype was not observed in EGFP-infected cultures (A, C). Neurospheres were immunostained with an antibody against cleaved caspase 3, which labeled very few cells in either EGFP or Gsx2-EGFP cultures (E, F). Scale bar (shown in F) = 338.9 µm (A–D); insets = 125.7 µm; 28.85 µm (E, F).

When we studied the capacity of E13.5 OBSCs to form neurospheres after Gsx2 overexpression, the Gsx2-EGFP infected neurospheres looked smaller than the control EGFP neurospheres 3 to 4 days after infection (Figure 2). Furthermore, while the control neurospheres floated in the medium, many of the Gsx2-EGFP neurospheres were attached to the plate, with individual cells lying outside the neurospheres (Figure 2 B). To test if the Gsx2 retrovirus caused apoptotic cell death, neurospheres were immunostained with an antibody against cleaved caspase 3. The small number of cells labeled for caspase 3 (Figure 2 E, F arrows) indicates that both EGFP and Gsx2-EGFP neurospheres were largely composed of healthy cells. Indeed, no EGFP cells in the neurospheres were labelled by the caspase 3 antibody on the first, and third days in culture, while only 0.01% were labelled on the second day in culture. Similarly, 0.00%, 0.00% and 0.03% (±0.02) of the Gsx2-EGFP cells were labelled by this antibody on the first, second and third days in culture, respectively.

**Figure 3:**
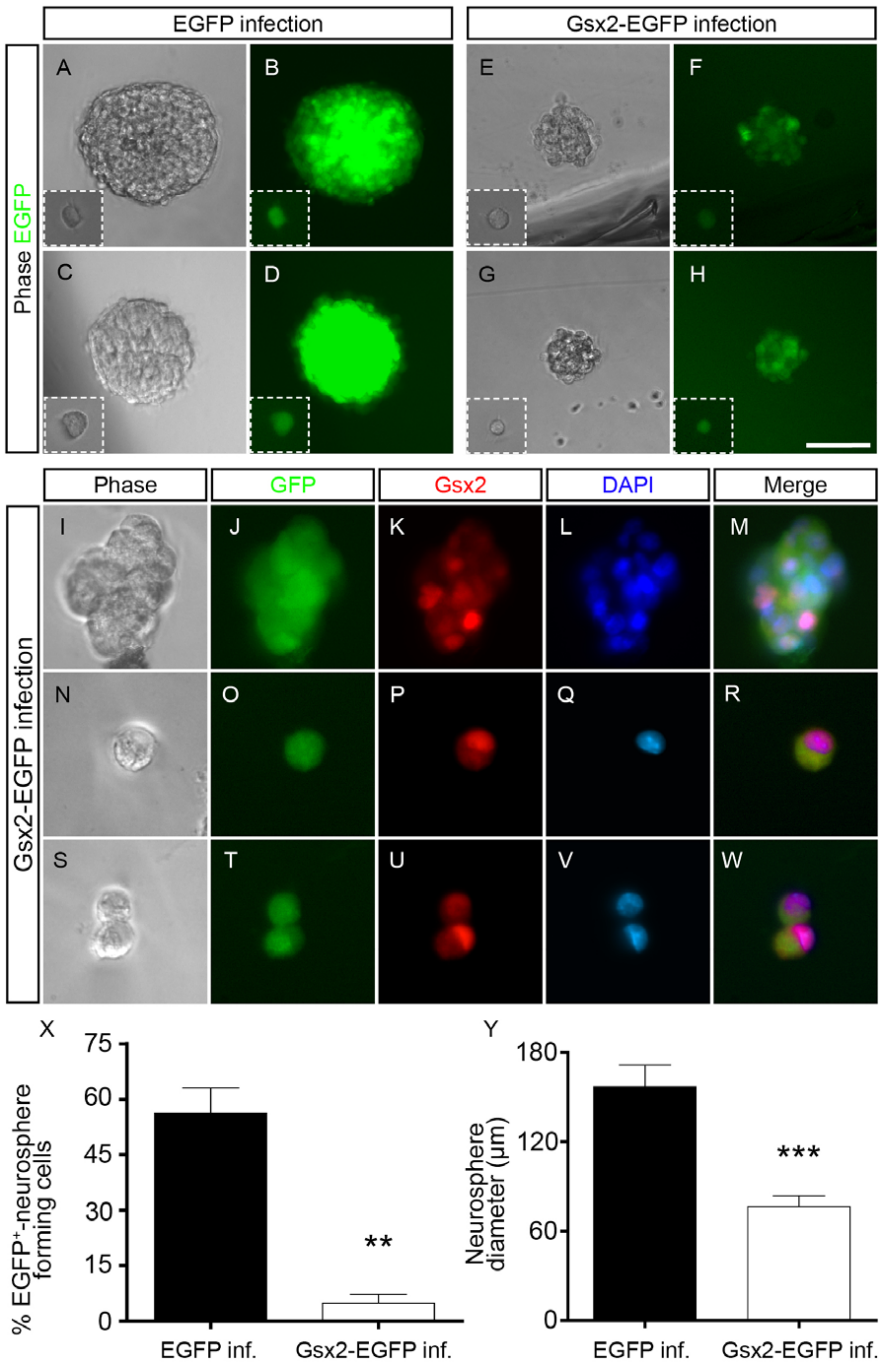
Gsx2 overexpression reduces the self-renewal capacity of NSCs. E13.5 OBSCs were cultured and passaged as floating neurospheres and then infected with the corresponding viral particles. Subsequently, single cells were plated for clonal analysis. The formation and growth of clonal neurospheres was visualized by phase contrast (A, C, E, G) and fluorescence microscopy (B, D, F, H). Two examples are shown for infection with each virus. Immunostaining revealed the Gsx2 expression in the transduced neurospheres (I–K) and DAPI staining indicates that Gsx2 mainly localizes to the nucleus (K–M). Some single cells and cell pairs were found in the Gsx2-EGFP clonal assay (N–W) but not in the control assay. The graph in X represents the mean percentage of single EGFP^+^ cells that formed neurospheres (mean ± s.e.m. from 3 experiments). The graph in Y represents the average sphere diameter (mean ± s.e.m., n = 33 and 21 neurospheres in EGFP and Gsx2-EGFP cultures, respectively). **P<0.01, ***P<0.001 versus values obtained from EGFP-infected cultures. Scale bar (shown in H) = 76.9 µm (A–H), 30.7 µm (I–M); 25.5 µm (N–W).

To determine whether Gsx2 affects NSC self-renewal and proliferation, we performed clonal analysis experiments, growing single E13.5 OBSC for 9 days. We found that 56.24% (±6.82) of the individual EGFP^+^ cells formed clonal neurospheres in control experiments (Figure 3 A–D, X) while only 4.79%±2.41 of EGFP^+^ cells formed neurospheres when they overexpressed Gsx2 (Figure 3 E–H, X). Moreover, the average diameter of Gsx2-EGFP neurospheres (76.46 µm±7.25) was half that of EGFP neurospheres (156.9 µm±14.56, P<0.0001: Figure 3 Y). Interestingly, we found single cells and pairs of cells in Gsx2-EGFP but not in EGFP cultures after 9 days of growth (Figure 3N, S). Dual immunostaining for Gsx2 and EGFP revealed that these isolated or paired cells co-expressed both proteins (Figure 3 N–W), similar to the cells that grew as neurospheres (Figure 3 I–M). Moreover, Gsx2 was primarily expressed in the nucleus, co-localizing with DAPI staining. Together, these data indicate that Gsx2 affects the normal growth of neurospheres and suggest that Gsx2-overexpression decreases the capacity of OBSC to self-renew and proliferate.

### NSC proliferation is affected by the presence of Gsx2

**Figure 4:**
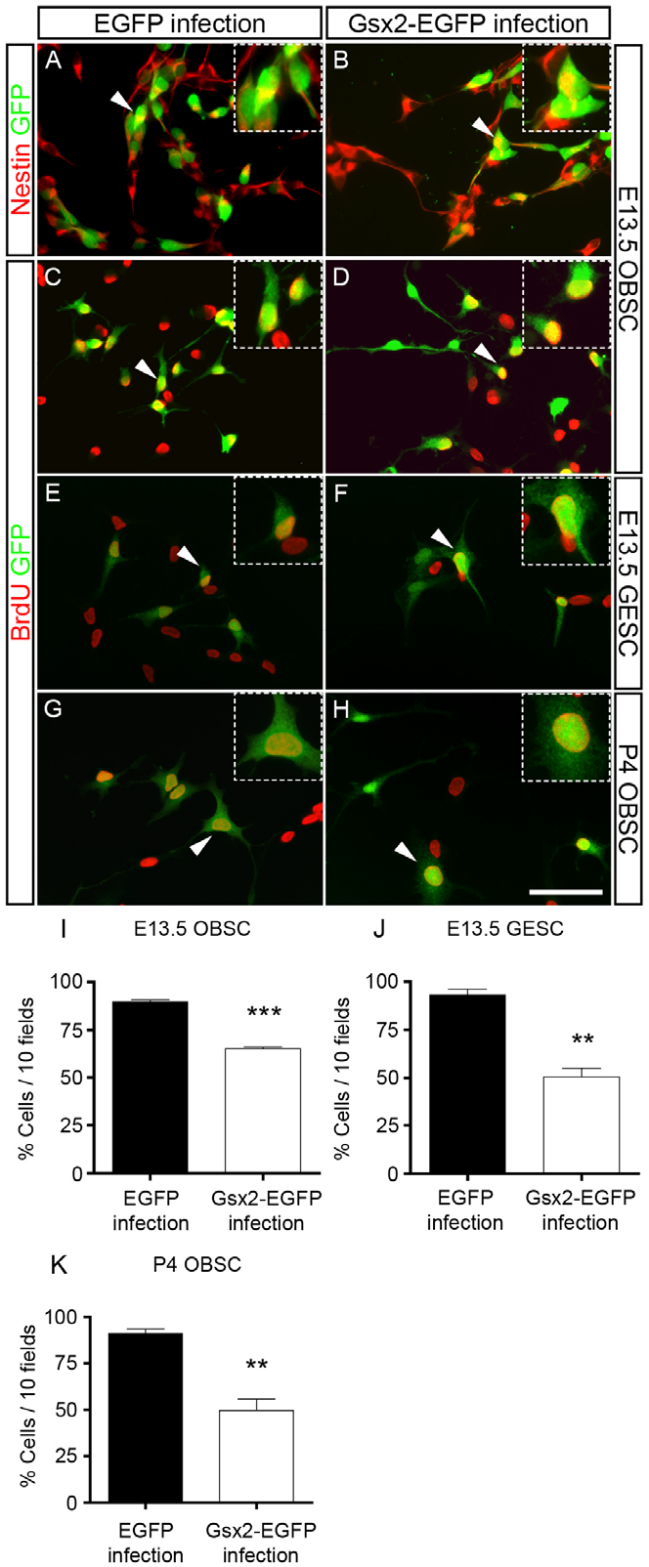
Effect of Gsx2 on NSC proliferation. Infected E13.5 OBSCs, E13.5 GESCs, and P4 OBSCs were grown in proliferation conditions for 3–4 days and labeled with BrdU 20 hours prior to fixation. Immunostaining for nestin and EGFP indicated that the vast majority of cells in both infected cultures are neuroepithelial cells (A, B). Immunostaining with antibodies against BrdU and GFP revealed that the majority of GFP^+^ cells in the controls had incorporated BrdU (C, E, G, I–K). Transduction of Gsx2 caused a 27–46% reduction in the cells labeled for BrdU^+^ and EGFP^+^ (D, F, H–K). The insets show high magnification images of the dual-labeled cells indicated with arrowheads in the main images. Graphs I–K represent the mean percentage (mean ± s.e.m., n = 3–6 cultures per condition). **P<0.01, ***P<0.001 versus the values obtained from EGFP-infected cultures. Scale bar (shown in H) = 59.5 µm; insets = 30.9 µm.

The reduced diameter of Gsx2-expressing neurospheres is consistent with the inhibitory effect of Gsx2 on cell proliferation. This was further investigated by growing cells for 3–4 days in the presence of FGF-2 and EGF, conditions in which the vast majority of Gsx2-EGFP-infected cells express the neuroepithelial marker nestin, as also seen in the control cultures (Figure 4 A, B). When proliferating E13.5 OBSCs were exposed to BrdU for the final 20 h in cultures, BrdU was incorporated into 89.68%±2.01 of the control EGFP-infected cells but only 65.24%±2.62 of the Gsx2-EGFP infected cells (a 27% decrease, P<0.0001: Figure 4 C, D, I). A marked effect of Gsx2 on BrdU incorporation was also observed when this transcription factor was overexpressed in E13.5 GESCs (45% reduction, P<0.01: Figure 4 E, F, J) and P4 OBSCs (46% reduction, P<0.01: Figure 4 G, H, K).

**Figure 5:**
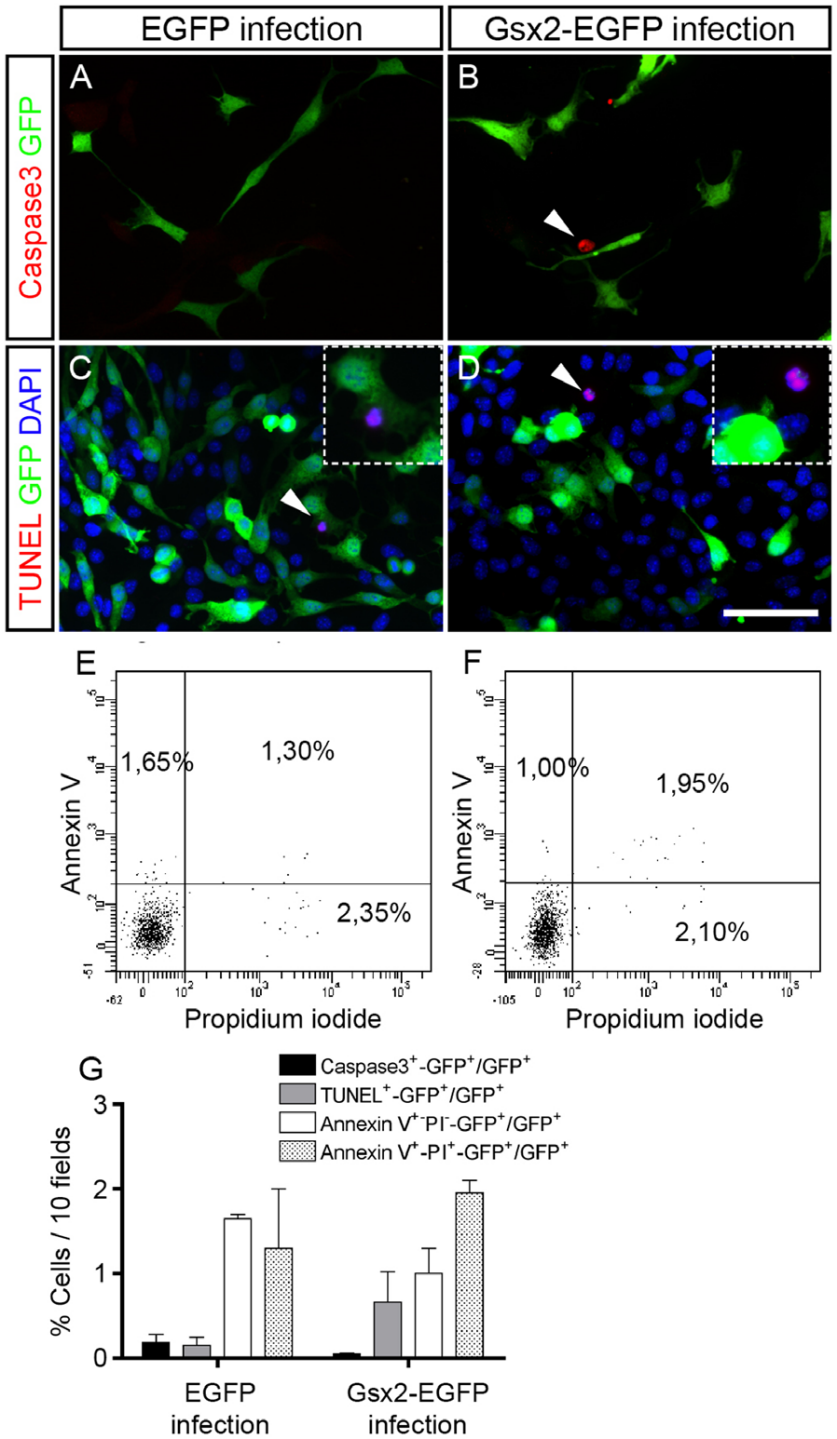
Effect of Gsx2 on cell death during the proliferation of NSCs. When cells were immunostained for cleaved caspase 3 (A, B), or subjected to a TUNEL assay (C, D) and counterstained with DAPI, there were very few apoptotic nuclei (arrowheads) in the cultures (A–D, G, n = 4 cultures per condition). The proportion of cells (measured by flow cytometry) that were APC annexin V^+^ and propidium iodide^−^ was between 0.25%–1.65% in EGFP cultures and between 0.30%–1.0% in Gsx2-EGFP cultures. Similarly, very few cells were APC annexin V^+^ and propidium iodide^+^ (E–G). Graph G represents the mean percentage (mean ± s.e.m., n = 3–4 cultures per condition). Scale bar (shown in D) = 59.5 µm; insets = 30.9 µm.

We then analyzed whether early and late cell death might influence the effects produced by Gsx2 overexpression in proliferating cells (Figure 5). Indeed, the proportions of EGFP cells (growing in adherent conditions) labeled with an antibody against cleaved caspase 3 were 0.22%, 0.59% and 0.19% during the first, second and third days in culture, while 2.13%, 0.63% and 0.01% of the Gsx2-EGFP cells were labeled by the antibody (Figure 5 A, B, G). Similarly, very few TUNEL^+^ cells were detected in the EGFP and Gsx2-EGFP cultures (0.15% and 0.66%, respectively: Figure 5 C, D, G). In addition, the proportions of cells that were APC annexin V^+^ and propidium iodide^−^ (an index of early apoptotic cells) were 0.25%, 0.45%, and 1.65% in EGFP cultures and 0.30%, 0.60%, and 1.0% in Gsx2-EGFP cultures when assessed by flow cytometry in the first three days after the corresponding passage. Likewise, very few cells were found to be APC annexin V^+^ and propidium iodide^+^ (an index of late apoptotic cells: Figure 5 E, F, G) at days 1, 2, and 3 (3.15%, 0.95%, and 1.30% in EGFP cultures and 0.95%, 3.10%, and 1.95% in Gsx2-EGFP cultures). These data indicate that the reduction in BrdU^+^ cells was not primarily due to an increase in cell death caused by Gsx2.

The reduced proliferation of NSCs could reflect the initiation of neuronal or glial differentiation. To test this possibility, we analyzed the expression of the following differentiation markers in cells grown in proliferation conditions: β-III-tubulin (TuJ1) for neurons, GFAP for astrocytes and O_4_ for oligodendrocytes. These markers were very difficult to detect in proliferating cultures of both EGFP and Gsx2-EGFP E13.5 OBSCs (Figure S2), suggesting that there was little differentiation under these conditions.

### Gsx2 overexpression diminishes the differentiation of NSCs into neurons and glia

**Figure 6:**
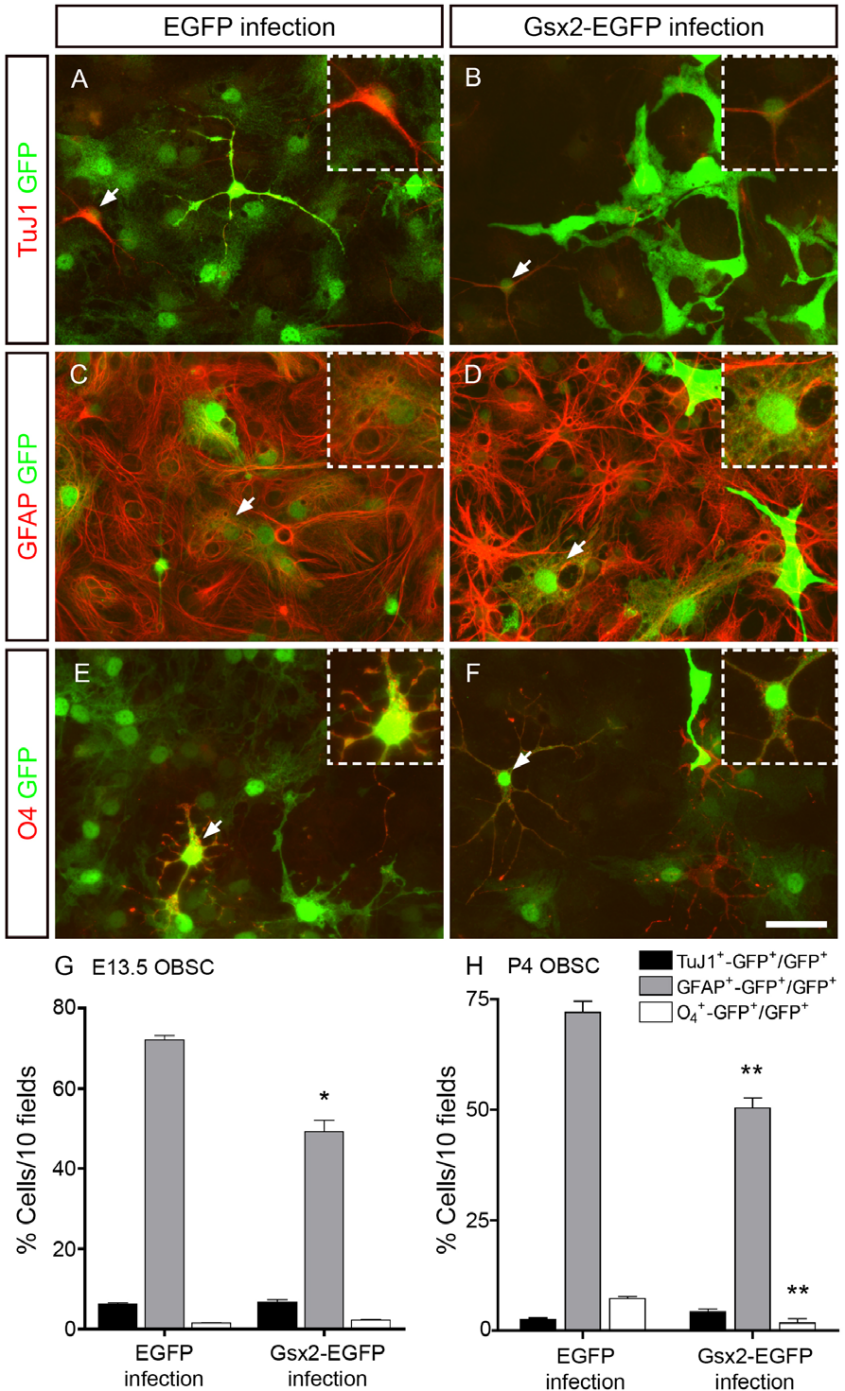
Transduction of Gsx2 decreases the capacity of OBSCs to differentiate into astrocytes and oligodendrocytes. E13.5 OBSCs transduced with viral particles expressing EGFP and Gsx2-EGFP were plated under differentiation conditions for 6 (not shown) and 20 days (A–F). Dual immunostaining with GFP and TuJ1, GFAP or O4 antibodies revealed the populations of neurons (A, B) and oligodendrocytes (E, F) to be similar in cultures infected with either virus. However, GFAP^+^-GFP^+^ cells were 32% fewer in the Gsx2-EGFP infected cultures than in those infected with EGFP alone (C, D, G). The insets show high magnification images of the double-labeled cells indicated with arrowheads in the main images. The graph in G represents the mean percentage ± s.e.m. (n = 8 cultures per condition; data from 6 and 20 days of differentiation were combined). *P<0.05 versus the values obtained from EGFP-infected cultures. P4 OBSCs transduced with viral particles expressing EGFP and Gsx2-EGFP were plated under differentiation conditions for 6 days (H). Dual immunostaining with GFP and TuJ1, GFAP or O4 antibodies revealed 30% GFAP^+^-GFP^+^ cells and 4-fold O_4_^+^-GFP^+^ fewer cells in the Gsx2-EGFP infected cultures than in the controls. The graph in H represents the mean percentage ± s.e.m. (n = 3 cultures per condition). *P<0.01 versus the values obtained from EGFP-infected cultures. Scale bar (shown in F) = 36.3 µm; insets = 21.7 µm.

We also investigated whether Gsx2 overexpression affected the cell fate determination and differentiation of NSCs. E13.5 OBSCs were differentiated for 6 (not shown) and 20 days (Figure 6 A–F) in the presence of 5% FBS and a single dose of FGF-2, as described previously [2]. Similar results were obtained at 6 and 20 days and as such, these data were combined in the analysis (Figure 6 G). Dual immunocytochemistry for GFP and TuJ1, GFAP and O_4_ revealed that a small proportion of the infected cells gave rise to TuJ1^+^ neurons or O_4_^+^ oligodendrocytes in both EGFP (TuJ1^+^, 6.29%±0.89; O_4_^+^, 1.46%±0.40: Figure 6 A, E, G) and Gsx2-EGFP (TuJ1^+^, 6.74%±1.67; O_4_^+^, 2.25±0.39: Figure 6 B, F, G) cultures. However, most GFP^+^ cells differentiated into astrocytes (GFAP^+^), although astrocytes represented a much smaller proportion of the population in Gsx2-EGFP cultures (49.17%±7.99; Figure 6 D, G: P<0.05) than in EGFP cultures (72.08%±3.02: Figure 6 C, G). The sustained expression of Gsx2 in differentiating P4 OBSCs caused a small increase in the percentage of TuJ1^+^ neurons that was not statistically significant (Figure 6 H). By contrast, Gsx2-EGFP cells were less capable of generating GFAP^+^ astrocytes (30% reduction; P<0.01) and O_4_^+^ oligodendrocytes (4-fold; P<0.01) than controls (Figure 6 H). Gsx2 overexpression in the medial ganglionic eminence was previously shown to drive cells towards a bipolar fate, with the cells expressing calretinin [26]. Thus, differentiated E13.5 OBSCs were immunostained for calretinin and GFP, and their morphology was analyzed. No significant differences were found in the number of bipolar calretinin-expressing neurons between EGFP and Gsx2-EGFP cultures (data not shown). Furthermore, no changes were apparent in the number of calbindin-positive neurons (data not shown).

**Figure 7:**
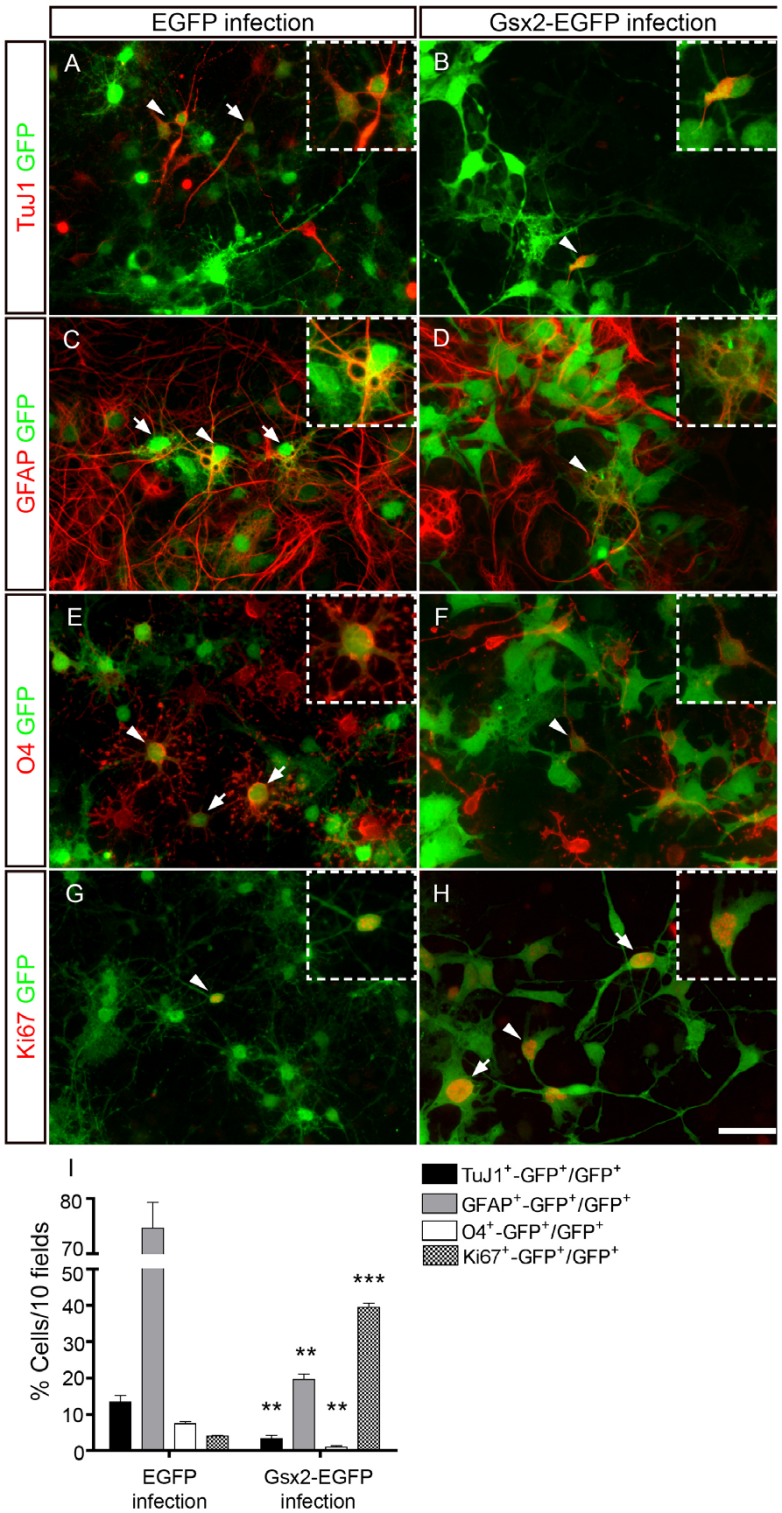
Transduction of Gsx2 decreases the capacity of GESCs to differentiate into neurons, astrocytes and oligodendrocytes. E13.5 GESCs transduced with viral particles expressing EGFP and Gsx2-EGFP were plated under differentiation conditions for 6 days. Dual immunostaining with GFP and TuJ1, GFAP or O4 antibodies revealed that the populations of neurons (A, B, I), astrocytes (C, D, I) and oligodendrocytes (E, F, I) were markedly reduced (3.8 to 8-fold fewer) in the Gsx2-EGFP infected cultures when compared to the controls. The proportion of infected cells labeled with an antibody against Ki67 was 9.7-fold greater in the Gsx2-EGFP infected cultures than in the controls (G–I). Arrows and arrowheads indicate GFP^+^ cells expressing a particular cell marker, and the insets show high magnification images of the double-labeled cells indicated with arrowheads in the main images. The graph in I represents the mean percentage ± s.e.m. (n = 3 cultures per condition). **P<0.01, ***P<0.001 versus the values obtained from EGFP-infected cultures. Scale bar (shown in H) = 36.3 µm; insets = 21.7 µm.

The influence of Gsx2 expression on the differentiation of NSCs was more pronounced when this transcription factor was introduced into E13.5 GESCs (Figure 7). Indeed, Gsx2-EGFP cultures had markedly fewer TuJ1^+^ neurons (4-fold; P<0.01), GFAP^+^ astrocytes (3.8-fold; P<0.001) and O_4_^+^ oligodendrocytes (8-fold; P<0.001) than EGFP cultures (Figure 7 A–F, I). This strong impairment in the ability of GESCs overexpressing Gsx2 to differentiate prompted us to further analyze the phenotype of cells growing under differentiation conditions. In these conditions, the proportion of Ki67^+^ cells (a general cell cycle marker) was 9.7-fold greater (P<0.001) in the Gsx2-GFP cultures than in the controls (Figure 7 G–I). In summary, our results show that high levels of Gsx2 can partially block the differentiation of NSCs, maintaining NSC-derived progenitors in the cell cycle. They also show that the magnitude of the effects triggered by Gsx2 is dependent on the specific NSC population in which it is expressed.

### Gsx2 overexpression affects the division of postnatal OB progenitors *in vivo*

**Figure 8:**
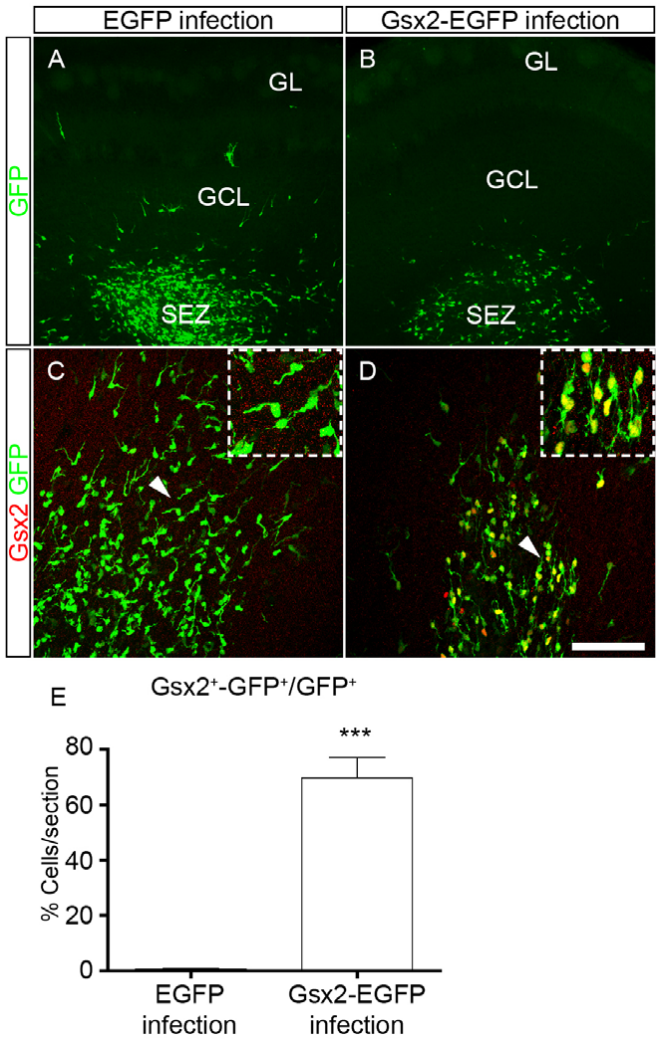
Injection of retroviral particles to express Gsx2 in dividing OB progenitor cells *in vivo*. Retroviral particles expressing EGFP or Gsx2-EGFP were injected into the OB subependymal zone (SEZ) of P4 mice to infect dividing progenitor cells. The mice were analyzed at P7, when GFP-labeled cells were mainly found in the SEZ. Low magnification images of OB coronal sections show the distribution of infected cells after immunostaining for GFP (A, B). Immunostaining for Gsx2 and GFP show that 69.85% of GFP^+^ cells expressed Gsx2 in animals injected with Gsx2-EGFP particles (D, E) compared to only 0.61% in animals injected with EGFP particles (C, E). The insets in C, D are high magnification images of the cells indicated by the arrowheads in the main images. The graph in E represents the mean percentage ± s.e.m. (n = 3 animals per condition) of the infected cells expressing both Gsx2 and GFP. ***P<0.001 versus the values obtained from EGFP-infected cultures. Scale bar (shown in D) = 42.2 µm (A, B), 8.65 µm (C, D); insets = 44.25 µm.

Having established that overexpression of Gsx2 reduces the self-renewal and proliferation of cultured NSCs, we studied the effect of Gsx2 on progenitors *in vivo* by injecting EGFP and Gsx2-EGFP retroviral vectors into the postnatal OB of mice (Figure 8). Viral particles were injected into the P4 OB subependymal zone (SEZ), given the presence of a considerable number of dividing progenitors in this region that do not express Gsx2 (see below; [2]). We found many GFP^+^ cells in the OB 3 days after injection, mainly located in SEZ, although labeled cells with a migratory morphology were also detected in the granule cell layer (GCL: Figure 8 A, B). Dual immunostaining for Gsx2 and EGFP revealed that 69.85%±7.30 of GFP^+^ cells co-expressed Gsx2 in Gsx2-EGFP infected mice (n = 3: Figure 8 D, E), while co-localization was minimal in control animals (0.61%±0.37, n = 3: Figure 8 C, E).

**Figure 9:**
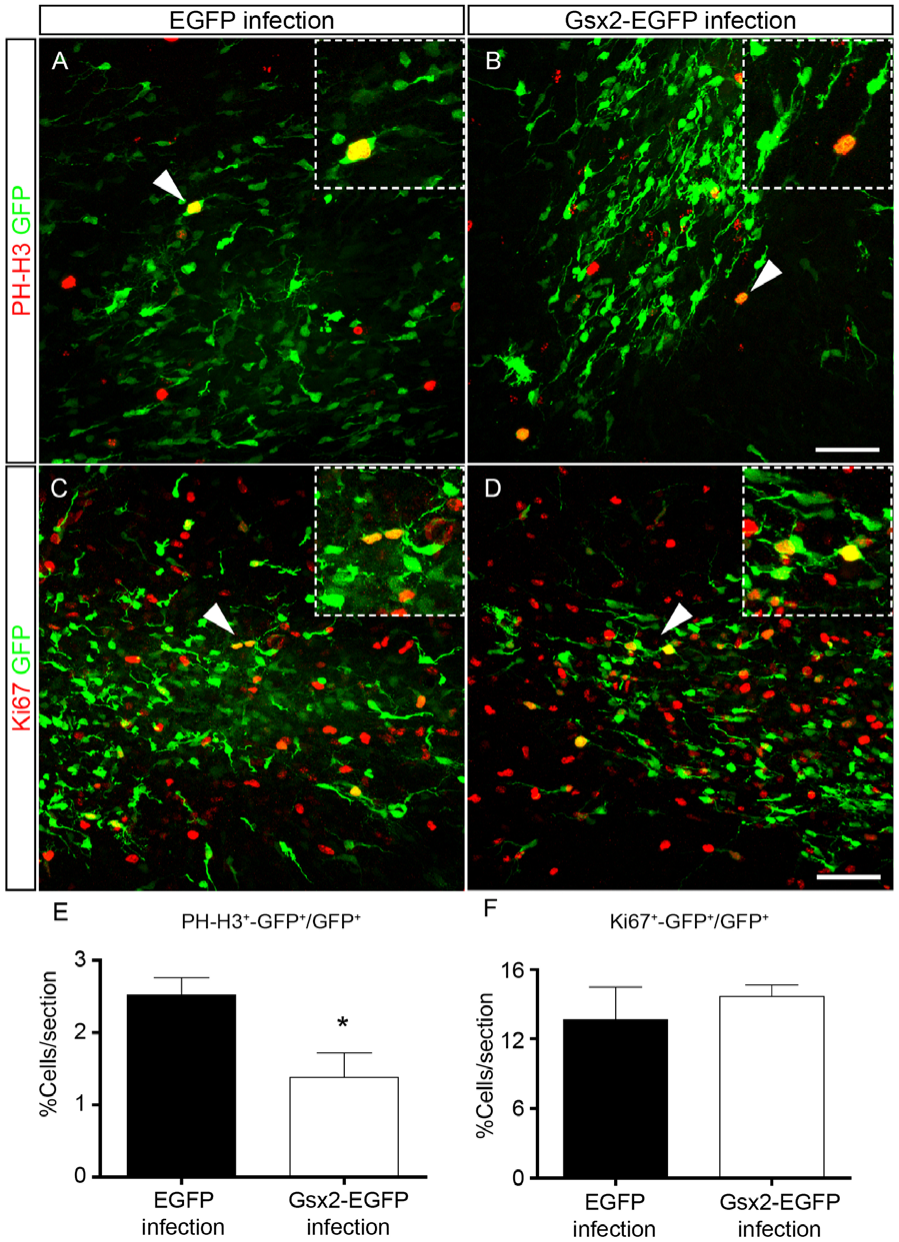
Effect of Gsx2 transduction on the proliferation of OB progenitor cells *in vivo*. Retroviral particles expressing EGFP or Gsx2-EGFP were injected into the OB SEZ of P4 mice. OB sections obtained at P7 were immunostained with antibodies against GFP and the mitotic marker Phospho-Histone-H3 (PH-H3) (A, B) or the cell cycle marker Ki67 (C, D). The insets show high magnification images of the double-labeled cells indicated with arrowheads in the main images. Gsx2 overexpression caused a 45% reduction in the proportion of progenitors in mitosis (E) when compared with the controls, whereas the total number of cycling cells (F) was not significantly affected. Graphs in E and F represent the mean percentage ± s.e.m. (n = 5 animals per condition). *P<0.05 versus the values obtained from EGFP-infected cultures. Scale bar (shown in D) = 55.1 µm; insets = 31.9 µm.

We investigated the proliferation of dividing progenitors in postnatal OB mice that had received the viral particles (Figure 9). Dual immunostaining for GFP and the mitotic marker Phospho-Histone-H3 (PH-H3^+^) revealed a statistically significant difference, whereby the Gsx2-EGFP infected OB contained almost 50% fewer cells (1.38%±0.34, Figure 9 B, E) than the EGFP infected OB (2.52%±0.23; P<0.05, Figure 9 A, E). However, the proportion of cycling progenitors labeled with an antibody against Ki67 was not significantly different in the OBs infected with either retrovirus. These results suggest that Gsx2 influences the capacity of progenitor cells to enter mitosis *in vivo*.

### The expression of cell fate markers is altered in postnatal OB progenitors transduced with Gsx2

**Figure 10:**
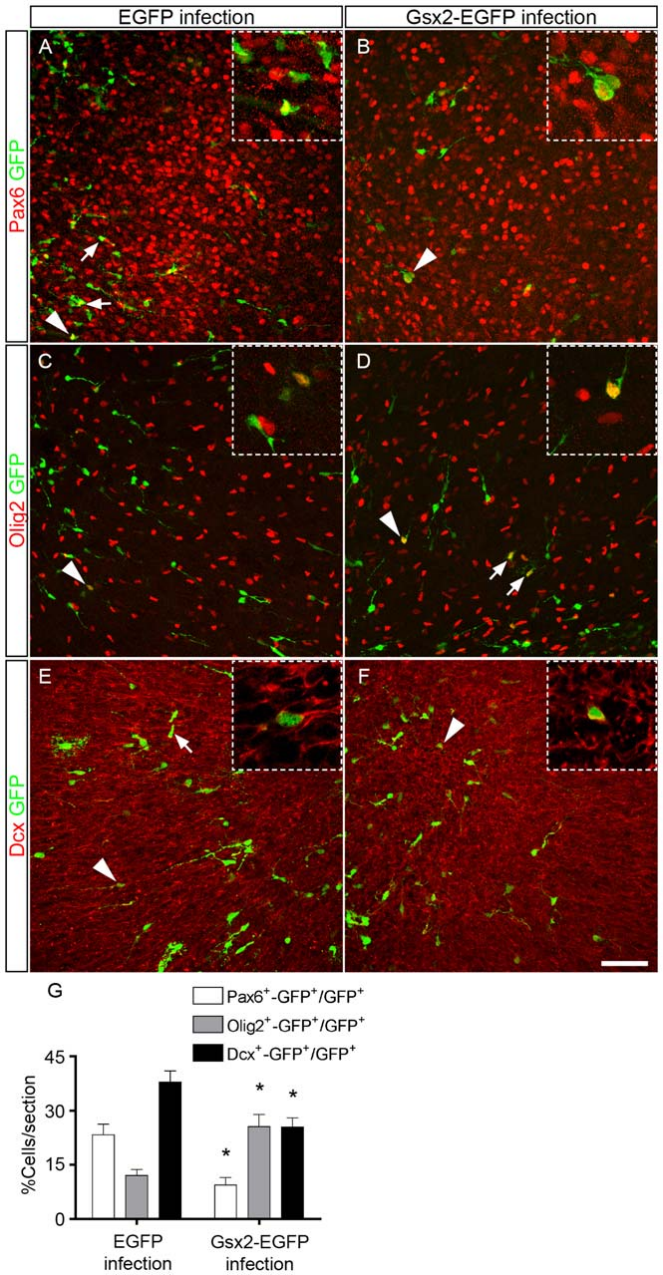
Transduction of Gsx2 changes the cell fate of postnatal OB progenitor cells *in vivo*. Retroviral particles expressing EGFP or Gsx2-EGFP were injected into the OB SEZ of P4 mice, and OB sections were obtained at P9 and immunostained with antibodies against GFP and Pax6 (A, B), Olig2 (C, D) or Dcx (E, F). Arrows and arrowheads indicate GFP^+^ cells expressing a particular cell marker, and the insets show high magnification images of double-labeled cells indicated with arrowheads in the main images. Gsx2 overexpression caused a 59.4% reduction (*P<0.05) in the number of Pax6^+^ expressing cells, a 110.4% increase (*P<0.05) in the number of Olig2^+^ cells, and a 33% decrease (*P<0.05) in the number of Dcx^+^ cells (G) when compared with controls. The graph in G represents the mean percentage ± s.e.m. of cells expressing GFP and Pax6 or Olig2 or Dcx (n = 3–4 animals per condition). Scale bar (shown in F) = 57.0 µm; insets = 23.4 µm.

Through the repression of dorsal genes such as *Pax6*, Gsx2 is thought to influence the establishment of the pallium-subpallium boundary in the telencephalon [9], [21], [22]. In addition to its role in dorso-ventral patterning, Pax6 is expressed in the rostral migratory stream (RMS) and in OB interneurons [32], [33], [34], [35]. To test whether Gsx2 has a direct effect on the identity of individual progenitor cells, we investigated whether transduction of Gsx2 altered the expression of Pax6 [36] and Olig2, a key transcription factor involved in the generation of oligodendrocytes in the subventricular zone/OB pathway that is abundant in the ventral telencephalon [33], [37], as well as that of Dcx, a marker of newly generated and migrating neurons [38]. In vibratome sections immunostained for Gsx2 and GFP 5 days after injection (P9) of the retroviral vectors into the P4 mouse OB (Figure 10), strong Gsx2 expression persisted in Gsx2-EGFP infected progenitors (63.13%±5.11 of GFP^+^ cells expressed Gsx2, while only 1.56%±0.74 did so in those infected with EGFP: n = 3 animals per condition). In control animals, 23.22%±2.92 of GFP^+^ infected cells co-expressed Pax6, 12.11%±1.59 co-expressed Olig2, and 37.84%±3.08 were positive for Dcx (Figure 10 A, C, E, G). By contrast, in Gsx2-EGFP infected progenitors there was a 2-fold decrease in the number of Pax6^+^ cells (9.43%±2.07, P<0.05, n = 3), a 2-fold increase in Olig2^+^ cells (25.49±3.47, P<0.05, n = 3), and a 33% decrease in Dcx^+^ cells (25.34±2.59, P<0.05, n = 4) that co-expressed GFP (Figure 10 B, D, F, G). These results indicate that the presence of Gsx2 partially represses the capacity of postnatal progenitors to differentiate towards the neuronal lineage.

## Discussion

### Gsx2 regulates proliferation, self-renewal and differentiation of neural stem cells

There is evidence that Gsx2 is involved in controlling the pallial subpallial boundary in the embryonic telencephalon. Deletion of the murine *Gsx2* gene causes *Pax6* expression to expand into the LGE territory [9], [21], [22], [23], whereas Gsx2 gain-of-function in transgenic mice regulates LGE specification by inhibiting the expression of dorsal markers and promoting the expression of ventral markers [9]. In the OB, deletion of Gsx2 reduces the number of GAD67^+^ interneurons and augments that of Tbr1^+^ projection neurons [9], [22], [23], [24], [25], [27]. However, no direct influence of Gsx2 on the formation of OB interneurons has been described and in addition, the role of Gsx2 in regulating the self-renewal and multipotentiality of NSCs has been little explored.

In this study, we aimed to elucidate the effect of Gsx2 on NSC and progenitor cells in the mouse OB and GE by promoting the sustained expression of Gsx2 with retroviral vectors. This approach enabled us to analyze the role of this transcription factor at the single-cell level, both in culture and in vivo. Under these conditions, overexpression of Gsx2 alters the normal growth of NSCs as neurospheres, reducing their proliferation and self-renewal capacity. Moreover, Gsx2 impairs the differentiation of NSCs in culture into neurons, astrocytes and oligodendrocytes, and it promotes the maintenance of NSC-derived progenitors as cycling, undifferentiated cells. The magnitude of these effects is dependent on the specific NSC population in question. In vivo, Gsx2 reduces the capacity of OB progenitors to produce neuroblasts and immature neurons. Accordingly, our findings suggest that the levels of Gsx2 regulate the self-renewal and differentiation capacity of NSCs, as well as the neurogenic potential of postnatal telencephalic progenitors.

The sustained expression of Gsx2 in NSCs inhibits their capacity to proliferate and self-renew. Indeed, fewer and smaller diameter clonal neurospheres were produced when Gsx2 was expressed persistently in E13.5 OBSCs, and there was a significant decrease in the proportion of BrdU^+^ cells in the three NSC populations studied (E13.5 OBSCs, P4 OBSCs and GESCs). These effects on cell proliferation are consistent with the in vivo findings that Gsx2 is able to reduce cell division in postnatal OB progenitors. By contrast, neither early or late cell death events, nor markers of differentiation, were detected in significant numbers in proliferating NSC cultures maintained in the presence of FGF-2 and EGF, suggesting that Gsx2 does not induce cell cycle exit or differentiation. We found that on the first day in culture, caspase 3^+^ cells represented 2.13% and 0.22% of the total number of adherent cells in the Gsx2-EGFP and EGFP conditions, respectively. These data might indicate that cell death contributed to the reduced size of the spheres in the Gsx2-expressing cultures. However, the proportion of labelled cells did not increase during the second (0.63%) and third day (0.01%) in culture. Moreover, the percentage of caspase 3^+^ cells in the neurosphere assays was almost negligible in both conditions: between 0.00%–0.03% on days 1–3 in Gsx2-EGFP neurospheres, and between 0.00%–0.01% in the EGFP neurospheres. The proportions of early and late apoptotic cells on days 1–3 were similarly low in the two conditions. Together, these findings suggest that the reduced size of Gsx2-EGFP neurospheres was primarily due to the reduced capacity of the cells to proliferate.

The decrease of cells expressing PH-H3^+^ (a mitotic marker) when Gsx2 is overexpressed in vivo occurs with little change in the number of cells expressing Ki67 (a general cell cycle marker), suggesting that the cells that do not enter mitosis in the presence of Gsx2 remain as cycling progenitors and/or that the length of cell cycle has been altered. In support of this conclusion, when GESCs are induced to differentiate there is a significant proportion of GESC-derived progenitors that are labeled with Ki67 and that do not differentiate into neurons or glia.

Together, our findings suggest that sustained expression of Gsx2 produces two effects: the inhibition of self-renewal and the proliferation of NSCs; and the increase in NSC-derived intermediate progenitors that do not exit the cell cycle or differentiate. This latter effect could mean that a large proportion of progenitors are retained in the G1 phase since this is a critical control point in the cell cycle [39]. These actions will produce a marked reduction in the capacity of NSCs and progenitors to generate neuronal and glial cells.

Our findings may help to understand the apparently contrasting phenotype observed in the LGE and cerebral cortex of Gsx2 knockout mice. Indeed, neurospheres derived from the LGE of Gsx2 knockout mice grow more slowly than control neurospheres, probably due to slight changes in their proliferative capacity [40]. Similar alterations in the maintenance of progenitor cell proliferation by Gsx2 have been observed in the subventricular zone of the LGE of Gsx2 mutant mice [25], and that of Gsx2 and Gsx1 double mutants [27]. By contrast, progenitor proliferation in the cerebral cortex is enhanced in Gsx2 knockout (KO) mice [30] whereas others [23], found no evidence that the mitotic index in the ventricular zone was affected in the Gsx2 KO. According to the results presented here, the influence of Gsx2 on NSC and progenitor proliferation, cell cycle progression and exit from the cell cycle, may depend on whether the cell is a NSC or an intermediate progenitor during the major waves of proliferation, neurogenesis and gliogenesis that occur in different brain regions and at distinct stages of development.

Such a concept could also reconcile our data and that which recently showed that Gsx2 misexpression augments the self-renewal capacity of progenitors isolated from the embryonic cerebral cortex and LGE [29]. By contrast, our clonal analysis indicates that Gsx2 overexpression strongly impairs the generation of clonal neurospheres from single OB-derived cells. Regional differences might arise in the response to Gsx2 since the effect of this transcription factor was apparently stronger in cells derived from the cortex than in those from the LGE [29]. However, we performed a clonal analysis by plating a single NSC per well [2], [18], rather than plating more heterogeneous cell populations at low density [29]. In addition, as opposed to analysing Gsx2 activity in transgenic mice and then in cell culture [29], we performed here gain-of-function experiments overexpressing human Gsx2 cDNA in culture and in the neonatal OB in vivo.

The direct influence of Gsx2 on neural cell fate and differentiation has been seen to promote the generation of bipolar calretinin interneurons from progenitors of the medial ganglionic eminence [26]. By contrast, there is no evidence that Gsx2 directly promotes the generation of OB neurons. Our results show that Gsx2 misexpression does not induce neuronal differentiation from any of the three NSC populations studied. Indeed, the proportions of TuJ1^+^ neurons, bipolar calretinin^+^ neurons and calbindin^+^ neurons (data not shown) in Gsx2-expressing cultures were either no different than in the controls (results from E13.5 OBSCs and P4 OBSCs) or markedly reduced (results from E13.5 GESC). Furthermore, sustained expression of Gsx2 significantly reduces astrocytogenesis from the three NSC populations, and it strongly decreased oligodendrocyte generation from P4 OBSCs and E13.5 GESCs. As mentioned above, these findings indicate that Gsx2 inhibits NSC differentiation, and that the magnitude of this effect is highly dependent on the region from where the NSCs were isolated and on the developmental stage in that particular region. The strongest effects of Gsx2 were observed in cells derived from the GE, primordial regions where Gsx2 is detected abundantly [8], [18], supporting the physiological relevance of the role described here for this transcription factor.

Although the molecular and genetic signalling pathways triggered by Gsx2 remain to be determined, these could involve the Notch signaling pathway that regulates self-renewal and gliogenesis [3], [28], the FGF-2 signaling pathway [18], the cross-repression of Gsx2 and Pax6 (as discussed above), and its interaction with other transcription factors including Gsx1 [29] and Nolz1 [41]. Nevertheless, our findings that Gsx2 may maintain NSCs and progenitors in an undifferentiated state concur with other recent findings where Gsx2 overexpression inhibited the neuronal differentiation of cortical and LGE progenitors, whereas the number of Sox2^+^ cells (a neural progenitor marker) increased [29].

The reduced self-renewal and differentiation potential of NSCs suggests that Gsx2 may be involved in the maintenance of these cells in a quiescent state. Indeed, single undifferentiated Gsx2-expressing cells persist in the clonal analysis, even in the presence of growth factors (FGF-2 and EGF), suggesting that other signals are necessary to activate self-renewal and/or differentiation. Among CNS progenitor cells, quiescence is a feature of adult NSCs [42], [43], [44] and it is notable that a considerable proportion of the NSCs located in the adult SVZ are derived from LGE-cells expressing Gsx2 [45]. However, it is unclear at present whether the existence of high levels of Gsx2 in subpopulations of LGE stem cells is part of the molecular machinery involved in establishing and maintaining them quiescent in the adult SVZ.

### Gsx2 regulates the differentiation potential of postnatal progenitor cells *in vivo*

The phenotype of Gsx2 knockout mice indicates that Gsx2 is necessary to maintain dorso-ventral patterning in the telencephalon [23], [24], [25], although there was no evidence that this transcription factor directly represses dorsal markers in these studies. However, misexpression of Gsx2 in the mouse embryonic telencephalon was recently shown to repress Pax6 and to up-regulate the ventral markers, Dlx and Ascl1 [9], indicating that Gsx2 is necessary to correctly specify the ventral identity of the LGE. Indeed, ind/Gsx2 overexpression dowregulates eyeless/Pax6 in the Drosophila embryo [46].

We show here that Gsx2 reduces the number of progenitor cells expressing Pax6 and Dcx in the early postnatal OB, coupled with an increase in cells expressing Olig2. In the postnatal-adult SVZ-OB, Pax6 specifies the differentiation of progenitors towards the production of neurons and it is required for the generation of specific populations of granule and periglomerular interneurons [33], [47]. By contrast, Olig2 counteracts the neurogenic action of Pax6 and promotes the generation of oligodendrocytes [33]. Our results demonstrate that ectopic Gsx2 can directly induce the partial specification of cell fate in dividing progenitors, reducing their capacity to generate neuroblasts (Dcx^+^ cells) that may possibly be destined to the interneuron lineage (Pax6^+^ cells). Accordingly, misexpression of Gsx2 in the dorsal telencephalon reduces the intensity of the neuronal marker β-III-tubulin [29], whereas an increase in the number of cortical neurons has been detected in *Gsx2* mutants [30].

The enhanced number of Olig2^+^ cells caused by the ectopic application of Gsx2 in the postnatal OB could indicate that this transcription factor favors oligodendrogenesis. Alternatively, since Olig2 promotes a transit-amplifying progenitor state in the SVZ-OB [33] and it is expressed by neural progenitors in culture [48], it cannot be ruled out that the enhanced number of Olig2^+^ cells in vivo might reflect an increase in the number of cells with the characteristics of progenitors, produced by Gsx2 overexpression. We attempted to resolve this question through an immunohistochemical analysis with O_4_ and Sox10 antibodies. However, the poor tissue penetration of our antibodies prevented us from determining whether Gsx2 promotes oligodendrocyte formation in vivo or not. Nonetheless, our in vitro results show a marked reduction in O_4_^+^ cells originating from P4 OBSCs and GESC overexpressing Gsx2, indicating that Gsx2 inhibits the formation of oligodendrocytes in culture.

All these results suggest that postnatal dividing cells are still competent to at least partially change their phenotypic identity in response to genetic cues, opening the possibility of re-specifying the differentiation potential of NSCs located in the adult SVZ-OB axis [18], [45], [49] using retroviral vectors expressing Gsx2.

In summary, we present here novel findings showing that Gsx2 regulates the quiescent and the undifferentiated states of NSCs and progenitors. This is achieved at two levels: through the inhibition of self-renewal and of the proliferation of NSCs; and by maintaining NSC-derived progenitors in the cell cycle. The final output is a marked reduction in the capacity of NSCs and progenitors to differentiate into neurons, astrocytes and oligodendrocytes. The magnitude of these effects depends on the developmental stage, and the region where the NSCs and progenitors are located. Our findings also support the notion that Gsx2 elicits a direct, cell-autonomous inhibition of neurogenesis from postnatal telencephalic progenitor cells.

## Supporting Information

Figure S1 Semi-quantitative PCR with specific primers was used to measure *Gsx2* and *Gapdh* expression. (A) Western blot to detect Gsx2 and β-tubulin in protein extracts obtained from transduced OBSC cultures. (B) A densitometric analysis of the data from three experiments is shown (C), expressing the results as the ratio of Gsx2 relative to ß-tubulin (mean ± s.e.m.). A marked increase in Gsx2 protein and mRNA is evident in samples from the Gsx2-EGFP-transduced cultures.
(TIFF)

Figure S2 In EGFP and Gsx2-EGFP-transduced proliferating E13.5 OBSCs cultures, immunostaining revealed virtual no TuJ1^+^ (β-III-Tubulin) neurons, GFAP^+^ astrocytes and O_4_^+^ oligodendrocytes. The same pattern was obtained in the 4 different cultures analyzed for each condition. Scale bar (shown in R) = 58.8 µm.
(TIFF)
