# Supplementary figures and images for: The Homeobox Gene Gsx2 Regulates the Self-Renewal and Differentiation of Neural Stem Cells and the Cell Fate of Postnatal Progenitors
Source: PLoS One. 2012 Jan 5;7(1):e29799. doi: 10.1371/journal.pone.0029799 (PMC3252334; doi:10.1371/journal.pone.0029799)

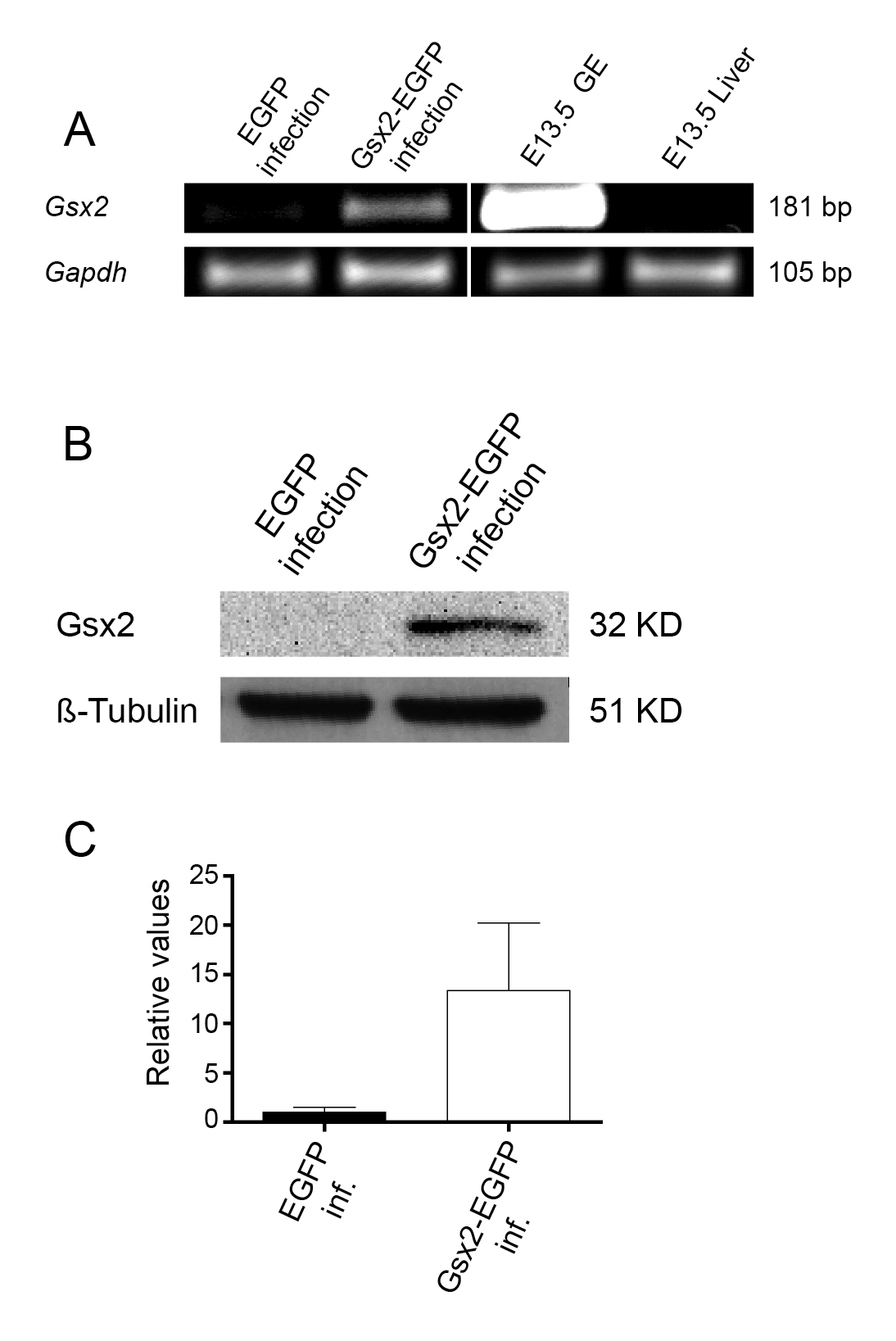

Supplement: Figure S1 — Semi-quantitative PCR with specific primers was used to measure Gsx2 and Gapdh expression. (A) Western blot to detect Gsx2 and β-tubulin in protein extracts obtained from transduced OBSC cultures. (B) A densitometric analysis of the data from three experiments is shown (C), expressing the results as the ratio of Gsx2 relative to ß-tubulin (mean ± s.e.m.). A marked increase in Gsx2 protein and mRNA is evident in samples from the Gsx2-EGFP-transduced cultures. (TIFF) [file pone.0029799.s001.tif]

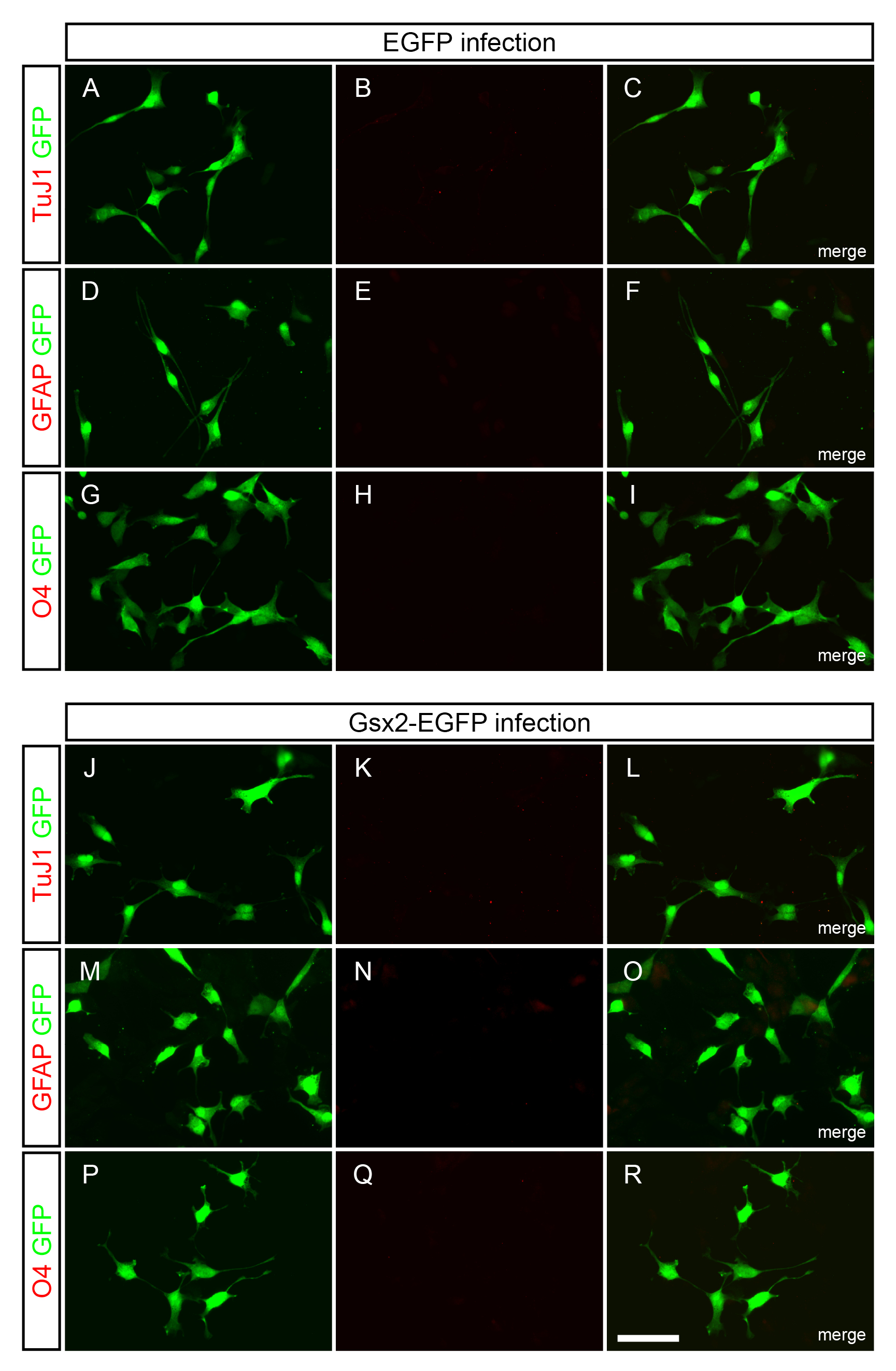

Supplement: Figure S2 — In EGFP and Gsx2-EGFP-transduced proliferating E13.5 OBSCs cultures, immunostaining revealed virtual no TuJ1+ (β-III-Tubulin) neurons, GFAP+ astrocytes and O4 + oligodendrocytes. The same pattern was obtained in the 4 different cultures analyzed for each condition. Scale bar (shown in R) = 58.8 µm. (TIFF) [file pone.0029799.s002.tif]
